# Outtakes from My Journey through the World of LIPID MAPS

**DOI:** 10.3390/molecules27123885

**Published:** 2022-06-17

**Authors:** Edward A. Dennis

**Affiliations:** 1 Department of Chemistry and Biochemistry, University of California at San Diego, La Jolla, CA 92093-0601, USA; edennis@ucsd.edu; 2 Department of Pharmacology, School of Medicine, University of California at San Diego, La Jolla, CA 92093-0601, USA

**Keywords:** lipid, lipid metabolism, LIPID MAPS, phospholipase, phospholipase A_2_, Keystone Symposia, Gordon Research Conferences, Journal of Lipid Research

## Abstract

My laboratory’s research on lipids has focused on phospholipases and lipidomics and in many ways has evolved in parallel to the evolution of the lipid field over the past half century. I have reviewed our research elsewhere. Herein, I describe the “side stories” or “outtakes” that parallel the main story that focuses on our laboratory’s research. I will emphasize the importance of community activities and describe how I came to initiate and lead the international effort on the Lipid Metabolites and Pathways Strategy (LIPID MAPS). Several of these side activities had a significant effect on discoveries in my laboratory research and its evolution as well as contributing significantly to the development of the LIPID MAPS initiative. These included experience and influences from serving as Editor-in-Chief of the *Journal of Lipid Research* and Chair and President of the Keystone Symposia on Cell and Molecular Biology as well as other experiences in organizing lipid conferences, teaching on lipid structure and mechanism, and earlier formative administrative and leadership experiences. The relevant influences are summarized herein.

## 1. Background

### 1.1. Prologue

Professor George Kokotos (Department of Chemistry, University of Athens, Athens, Greece) and Professor Jesús Balsinde (Institute of Molecular Biology and Genetics, University of Valladolid, Valladolid, Spain) informed me that they were serving as editors for a Special Issue of *Molecules* honoring my career and invited me to contribute a review. Because I have already thoroughly reviewed our group’s scientific research (see below), I will only provide a short summary of my research trajectory (next section). Rather than writing another “*reflection*” on my research per se, I will describe the side stories or “outtakes”; that is the way seemingly peripheral community interests and activities have had a major influence on my scientific research and particularly in leading the LIPID MAPS initiative aimed at developing global lipidomics.

### 1.2. Research Focus Summary

Technically, my academic research has focused on the enzyme phospholipase A_2_ (PLA_2_) for nearly half a century. However, PLA_2_ has merely been a vehicle for studying enzyme mechanism, protein–lipid interactions, cell signaling, inflammation, lipidomics, inhibitors, and the initiation of the immunologically derived inflammatory response that accompanies most of the diseases of our times. This includes interest in and application of lipidomics to metabolic syndrome and its hallmarks of obesity, insulin resistance and type 2 diabetes, atherosclerosis and a large variety of cardiovascular diseases, and the latest manifestation of metabolic syndrome, namely nonalcoholic fatty liver disease (NAFLD) and its progression to nonalcoholic steatohepatitis (NASH) and cirrhosis of the liver. My special interest has focused on the inflammatory component of all of these illnesses.

A decade ago, our group, in collaboration with former sabbatical visitor Professor George Kokotos, provided an “encyclopedic” review of the field of phospholipase A_2_ enzymes and their inhibition with over 500 references to the work carried out by other scientists around the world, as well as our own contributions [1]. In 2015, graduate student Paul Norris and I summarized the central role of PLA_2_ in eicosanoid biology and inflammation [2]. In 2016, in response to an invitation from Herb Taber, Editor of the *Journal of Biological Chemistry* (*JBC),* to write a *“Reflection*” on my scientific career to date, an article entitled “Liberating chiral lipid mediators, inflammatory enzymes and LIPID MAPS from biological grease” resulted [3]. More recently, my ASBMB Vallee Award Lecture appeared in *JBC*, and this included an update on PLA_2_ lipidomics and mechanism [4]. Together with Varnavas Mouchlis, a former postdoctoral and project scientist, we have recently reviewed the current state of PLA_2_ specificity and the role of allosteric interactions with membranes (V. Mouchlis and E. A. Dennis (2022), *Accounts of Chemical Research*, in preparation).

Regarding our lipidomics efforts and the development of LIPID MAPS, the consortium’s early vision was summarized in 2005 [5] as well as our laboratory’s early approach to eicosanoid lipidomics [6]. LIPID MAPS’s further evolution and organization [7] and our laboratory’s basic approach to quantitatively analyzing eicosanoids [8] and fatty acids [9] were summarized in 2008–2009. With Oswald Quehenberger, we described the potential of lipidomics in the analysis of blood in 2010 [10], and I included some of LIPID MAPS specific accomplishments in the *Reflection* [3]. Others have also commented on LIPID MAPS development and impact [11,12,13] and the broader development of lipidomics [14]. The evolution of LIPID MAPS to its present status under the leadership of Professor Valerie O’Donnell (Cardiff University, United Kingdom) and financial support from the Wellcome Foundation (UK) along with the further development of lipidomics is described elsewhere [15].

### 1.3. Scientific Journey across Disciplines and Technologies Leading to LIPID MAPS

From a technology point of view, in college at Yale University, I focused on organic chemistry and benefited from valuable research experience in synthetic organic chemistry with Professor Harry H. Wasserman in the Department of Chemistry supported by NSF Summer Research Fellowships. In graduate school in the Department of Chemistry at Harvard University, I received an NIH Graduate Fellowship. My doctoral thesis with Professor Frank H. Westheimer on “Pseudorotation in Phosphate Ester Hydrolysis” was in physical organic chemistry, which included using nuclear magnetic resonance (NMR) spectroscopy and mass spectrometry (MS), which were in their infancy, and these tools later became critical to my research on micelles and phospholipases as well as in lipidomics as part of LIPID MAPS. As a postdoctoral fellow with Professor Eugene P. Kennedy at the Harvard Medical School’s Department of Biological Chemistry, NIH supported me with an Individual Postdoctoral Fellowship. This was my first research experience with biochemistry and cell biology, but in particular, in encountering genetic approaches to lipid metabolism and enzymology.

As a new independent faculty member in 1970 at the University of California, San Diego in the Department of Chemistry (later renamed the Department of Chemistry and Biochemistry), I was at last free to do “whatever I wanted to” research-wise. I set out to study membrane–lipid–protein interactions using as a prototype a water-soluble enzyme that catalyzed an enzymatic reaction on a phospholipid molecule that was part of a membrane bilayer as the essence of protein–lipid interactions. Here, we could observe and quantify a biological effect at the molecular level. I then sought to identify an enzyme that could fulfill that role. Eventually, I settled on phospholipase A_2_ as the ideal enzyme for these studies once I learned that we could purify this enzyme in large quantities from snake venom.

Over time, these studies naturally led me to the fields of enzyme kinetics, mechanism and inhibition. I wanted to study more accessible forms of the “lipid–water interface” than bilayer membranes, especially micelles and mixed micelles with surfactants; this led me to become more of a colloid and surface scientist using NMR techniques to examine phospholipid interactions and protein–lipid interactions. Later, when I recognized that PLA_2_ was the key trigger for generating free arachidonic acid for prostaglandin biosynthesis and the biosynthesis of related inflammatory mediators, I became interested in the eicosanoid field and biochemical consequences of inflammation. This led me to develop more as a full-fledged biochemist, mastering enzyme purification, mechanism, and metabolic pathways.

In 1983, I took my first sabbatical leave as a Guggenheim Fellow during which I learned to work with mice and harvest macrophages as a visiting professor with Professor Manfred Karnovsky at Harvard Medical School, a noted pharmacologist/physiologist/biochemist. I also learned how to carry out radioimmune assays (RIA) on eicosanoids as a visiting scientist with Professor Laurie Levine, a noted immunologist/biochemist at Brandeis University. With those new techniques in hand, my laboratory turned to more immunological techniques and cell biological approaches as well as a focus on eicosanoid chemistry and biochemistry with a special focus on macrophages, which play such a central role in immunological responses and inflammatory sequelae. Early studies on macrophage inflammation were in collaboration with immunologist Richard Ulevitch at The Scripps Research Institute (now Scripps Research).

Later, advances in mass spectrometry led us to develop the use of hydrogen–deuterium exchange mass spectrometry (HD/MS) in collaboration with the late Professor Virgil Woods (University of California, San Diego) to directly examine the interactions of PLA_2_ enzymes with membrane phospholipids [16]. This experimental approach naturally led to collaboration with Professor Andrew McCammon (University of California, San Diego, CA, United States of America) on applying molecular dynamics (MD) simulations to understanding and visualizing the results of HD/MS and explaining the mechanistic details of PLA_2_ action on each enzyme’s unique phospholipid substrate. These approaches allowed for further evolution of our exploration of PLA_2_ as a prototype for protein–lipid interactions at a much more fundamental level [4]. The power of MD to visualize PLA_2_ interactions with the catalytic machinery in the active site furthered our collaborations on inhibitor design and synthesis [17] with Professor George Kokotos (University of Athens). Furthermore, with the integration of lipidomics into the study of PLA_2_, this also led to a rekindled interest in inhibitors as therapeutic targets [18].

## 2. Lipid Maps Outtakes

### 2.1. Organizational Leadership of the LIPID MAPS Initiative

In 2002, after completing a three-year term as chair of the Department of Chemistry and Biochemistry, I applied for a planning grant for a proposal entitled “**LIPID M**etabolites **A**nd **P**athways **S**trategy”, now known by the shorthand “LIPID MAPS”, for a Large Scale Collaborative Grant (known as the GLUE grant program) at the National Institute of General Medical Sciences (NIGMS). This proposal included twelve co-principal investigators representing seven universities and a biotech company, and I served as the Principal Investigator. When we received the grant award in 2003, genomics was well established, as was proteomics, and this grant was specifically awarded to develop the “lipidomics” field; we received over USD 73 million during the next ten years to fulfil this mission. This brought me back to lipids, including their structural chemistry, organic synthesis and characterization, as well as their biological and biochemical function, cellular role, and disease implications. The development of lipidomics most significantly depended on new developments in mass spectrometry, which caused me to transition entirely from acquiring NMR spectrometers to mass spectrometers (MS). Han and Gross [19] have recently reviewed the evolution of mass spectrometry technologies in the service of lipidomics during this period.

Developing the field of lipidomics was not a solitary process; it required bringing a diverse group of scientists together to create a new interdisciplinary area. As important as the individual scientific contributions and discoveries were, it was equally important that the scientists work together in an interdisciplinary environment to evolve lipidomics. In hindsight, I drew on the many past scientific experiences described above as well as community activities described in subsequent sections to recruit expertise in the areas needed and, importantly, to select outstanding individuals who were the leading experts in their field, but who, I felt, could work together to create a functional organization and discipline.

Before development of the final grant application in 2002, we began by holding two weekend retreats in which we debated the multitude of ways to develop the field and to make choices among the myriad of possible directions. The first thing I did upon receiving the grant award notice was purchase an elaborate state-of-the-art video conferencing system with a setup for each of the twelve lead investigators; video conferencing was still new at that time and was expensive, but I felt it was essential if LIPID MAPS was going to succeed with its mission. During all of LIPID MAPS, we continued with both biannual in-person and bimonthly video conferencing discussions.

### 2.2. Development of LIPID MAPS Classification, Nomenclature and Structural Drawing

Early on, we recognized the need for a consistent and comprehensive classification system for lipids, as none existed. Although various classification systems existed for some classes of lipids, they were often not consistent with one another. As we proceeded to classify lipids, we quickly recognized the need for a consistent nomenclature that rigorously followed chemical naming rules to describe our classification system. Even more critical was the fact that chemists draw chemical structures and, again, while conventions had evolved for various classes of lipids, they were often different between classes. Thus, before we felt ready to begin lipidomics experiments, we devoted ourselves to developing a comprehensive classification, nomenclature, and structural drawing system that was inclusive of all lipids.

This initial digression proved important to the development of international cooperation among the leading lipid scientists across the world. The LIPID MAPS Consortium decided that it was crucial to involve the international community if it was to succeed in broad acceptance and use of one classification system. On a trip to Germany, I arranged a critical meeting with Kai Simons, Gerrit van Meer, and Fritz Spener in Dresden to plan how to engage the broader international lipid community. Eventually, I convinced two European and two Japanese scientists to work with the LIPID MAPS Consortium and to coauthor the initial paper with the LIPID MAPS Co-Investigators [20]. This led to the formation of the International Lipid Classification and Nomenclature Committee (ILCNC) composed of the authors of the initial paper. The ILCNC evolved its membership and continues to meet periodically to this day, and it produced an update of the system in 2009 [21] and again in 2021 [22] with Professor Fritz Spener (University of Graz, Graz, Austria), taking the lead under the auspices of the current LIPID MAPS Initiative supported by the Wellcome Trust [15].

Of special interest to me was developing and including the structural drawing program. Having taught lipid metabolism to undergraduates, graduate students, and first-year students in the School of Medicine at the University of California, San Diego, for many years, I became aware of how difficult it was to explain sphingolipids structurally in their relationship to phospholipids. This was especially true when trying to explain enzymatic pathways and reactions involving the inter-conversion of sphingolipids and phospholipids, and I had developed a stereo-chemically consistent way to draw both classes interacting. Dr. Eoin Fahy in the LIPID MAPS Bioinformatics Core worked hard at utilizing this approach. LIPID MAPS succeeded in creating and developing a fully consistent structural drawing approach and in incorporating it into the classification and nomenclature system.

Some thirty years earlier, I had become acutely aware of the challenges of chemical nomenclature when, in the early 1970s, Sidney P. Colowick and Nathan O. Kaplan recruited my wife and me to introduce the use of computers to produce an automated cumulative index for the *Methods in Enzymology* series. This series, which was central to biochemists, had grown to 30 volumes and lacked an integrated index; today, there are 669 volumes published. We discovered that even the American Chemical Society’s *Chemical Abstracts* had not defined chemical nomenclature rules that could order complicated chemical names that include alpha-numerics, Greek letters, Roman numerals, subscripts, superscripts, primes, + and − signs, various stereochemical notations, symbols, and other characters and phrases. My wife Dr. Martha Dennis, a computer scientist, designed such a system, which allowed Academic Press, Inc. to produce several cumulative indexes for *Methods in Enzymology* [23,24,25]. The rules we created for ordering chemical names are listed in the Preface and Notes to each volume.

The above experiences gave me a great appreciation of the importance of classification, nomenclature, and structural drawing when LIPID MAPS faced similar challenges. The ILCNC continues to meet periodically to advise the LIPID MAPS curators as new lipids are discovered. The LIPID MAPS Structural Database (LMSD) currently contains over 47,500 unique lipids (https://lipidmaps.org, accessed on 12 June 2022).

### 2.3. Scientific Leadership of LIPID MAPS

With regard to lipidomics itself, the most important choice we made after considerable debate with the LC/MS Core led by Professor Robert C. Murphy (University of Colorado, Denver, CO, USA) and which became our guiding principle was that we would create a lipidomics platform based on targeted approaches, including a rigorous quantitative system for lipidomics rather than qualitatively identifying molecules present. This required the development of numerous pure “primary standards” as well as numerous “internal standards”, the latter generally containing deuterium or occasionally containing fatty acids with an odd number of carbon atoms.

The Lipid Synthesis/Characterization core under the direction of Dr. Walter Shaw, President of Avanti Polar Lipids (Alabaster, AL, USA), was a critical component of the LIPID MAPS Initiative. It was led by a scientist who was the head of a private company but was crucial to the success of LIPID MAPS because this core could produce the necessary standards for quantitative mass spectrometric analysis, which included both primary standards and internal standards.

Early on, LIPID MAPS decided to focus lipidomics analysis on both blood (eventually settling on plasma) and on a single-cell type grown in cell culture, in particular the macrophage as the most representative prototypic mammalian cell. In order to develop consistency in the lipidomics experiments, a great effort was placed on developing one cell line that could be grown reproducibly. Further exploration by the Macrophage Biology Core led by Professor Christopher K. Glass (University of California at San Diego, La Jolla, CA, USA) resulted in a focus on the RAW 264.7 mouse macrophage cell line. A fresh aliquot of cells was obtained from the American Type Culture Collection (ATCC) and one thousand separate vials were stored in liquid nitrogen to last for a decade. A carefully selected lot of endotoxin-free fetal calf serum was purchased to last five years. The Consortium set out to produce a chemically homogeneous essential component of lipopolysaccharide (LPS) that could reproducibly be produced by the Synthesis Core. This resulted in the production of KDO_2_-Lipid A [26] under the direction of the late Professor Christian R.H. Raetz (Duke University, Raleigh, NC, USA).

Another early goal was to integrate the developments of lipidomics with the other omics, especially genomics; thus, there was an active interest in carrying out transcriptomics on the same systems as lipidomics for which the Bioinformatics Core under the direction of Professor Shankar Subramaniam (University of California at San Diego, La Jolla, CA, USA) integrated the omics analysis, particularly on the RAW cell studies [27].

The LIPID MAPS Consortium devoted its initial five years of funding to developing the systems, reagents, and approaches using a deliberately interactive, consultative process in order to optimally determine how best to lead the development of lipidomics for its long-term goals rather than short-term accomplishments. Consequently, it was not until after its renewal in 2008 that LIPID MAPS published its first comprehensive lipidomics analyses, but they represented integrated comprehensive approaches that involved all of the Cores and most of the investigators. The first comprehensive lipidomics paper was on the subcellular distribution of lipids in the RAW macrophage [28]. This was followed by a complete lipidomics analysis of the RAW cells and the changes induced by activation with LPS, specifically KDO_2_-Lipid A, and which included all six categories of mammalian lipids [29]. Importantly, at about the same time, the consortium completed its complete lipidomics analysis of human plasma, including the lipids in each of the six categories of mammalian lipids [30]. Its final integrated effort was aimed at developing lipid biomarkers for non-alcoholic fatty liver disease (NAFLD) and non-alcoholic steatohepatitis (NASH) and integrating them with other omics approaches [31].

## 3. Related Outtakes

### 3.1. Outtake: Early Interests

My interests in scientific research predated college. When I was a high school sophomore in Chicago, my biology teacher encouraged me to develop a science fair project, but I had no interest in dissecting frogs nor could I stand the sight of blood. However, I developed an apparatus to measure air pollution, not a popular subject back in the 1950s. It consisted of an old bicycle pump that I reversed to pump a vacuum in a liter jar equipped with a few repurposed plumbing valves. When released, it would suck a liter of air in through a tiny plastic orifice onto a microscope slide, where dust particles would adhere and I could count them. On a windy Chicago day, I would stand on a busy street corner downwind of the traffic, and determine the number of particles per liter emitted by the passing traffic compared to standing on the other side of the street. My project won a special award at the Chicago Science Fair in 1958 sponsored by the US Steel Corporation. It included a first-class trip accompanied by my chemistry and biology teachers to Pittsburg (the first airplane ride for all of us!) to tour the company’s research laboratories and to see how Pittsburg had stopped burning coal and recently recovered from its decades of air pollution. The visit included showing my apparatus to the then President of US Steel Corporation to demonstrate how the apparatus worked: I blew cigarette smoke in the air in order to capture the yellow tar on the slide, which could easily be seen without a microscope.

However, in high school, I was equally interested in the community and was involved in numerous high school activities. I was elected President of the student body at Senn High School, a large public high school. As president, I borrowed new voting machines from Major Daley’s City of Chicago for future school elections; thus, they would be more honest. Among my activities, I participated in the American Junior Red Cross, an organization devoted to helping children around the world. One consequence was that I was selected to join a group of 23 students from across the US to join a three-month “goodwill” tour of similar organizations throughout Europe. This was my first exposure to international relations, travel, and most importantly, working with people from other cultures and countries for the common good. This theme still applies to my scientific collaborations as well as with LIPID MAPS, creating a truly international lipidomics effort.

### 3.2. Outtake: Scientific Publishing

I became interested in organic chemistry as an undergraduate at Yale University, but during my scientific research career, it was understanding enzyme mechanism that most fascinated me. As an undergraduate, I became involved with the *Yale Scientific Magazine*, the nation’s oldest college science publication now in its 128th year, and served as its Chairman (equivalent to Editor-in-Chief) my senior year. As such, I received a lot of bulk mail including scientific books from publishers. One in particular caught my attention: it was a book on enzyme mechanism by Edward M. Kosower [32] describing how enzymes worked to catalyze chemical reactions (see book review by Myron Bender, then, one of the leading organic chemists studying enzymatic mechanisms [33]). Enzyme mechanism as a field was in its infancy, and the concept fascinated me, but I knew nothing about enzymes, having never taken a biology or biochemistry course in college. I think this fortuitous exposure contributed to my later choosing to carry out my thesis work with Professor Frank Westheimer at Harvard. Many consider him to be the “father” of enzyme mechanism (I only learned much later that Kosower and Bender had both been postdocs with Westheimer, as had Daniel Koshland, another great pioneer in studying enzyme mechanism), although I did not work on enzymes until after leaving his laboratory.

As a faculty member at the University of California, San Diego, I was elected to membership in the American Society of Biochemistry and Molecular Biology (ASBMB) where I served on the membership committee and as an Editorial Board Member of the *JBC*, the major journal in my field. After serving as Program Chair for the Annual Meeting and then Chair of the Annual Meetings Committee, I was elected to serve on the Publications Committee and eventually as its Chair during which time the Society started its first new journal *Molecular Cellular Proteomics* with Professor Ralph Bradshaw (University of California at Irvine, Irvine, CA, USA) as its Editor. It was also during that period that the ASBMB acquired the well-established and leading journal in the lipid field, the *Journal of Lipid Research* [34], from the non-profit Lipid Research Inc. (LRI), which was its publisher. I was invited to serve as the first Editor-in-Chief under ASBMB ownership, which I did for fifteen years. I asked Professor Joseph L. Witztum (University of California at San Diego, La Jolla, CA, USA) to serve as Deputy Editor and, after the first five-year term, invited him to join me as Co-Editor-in-Chief until he stepped down and Professor William Smith (University of Michigan, Ann Arbor, MI, USA) succeeded him. We along with the Associate Editors worked hard to integrate the advances in lipidomics into the evolving field of lipid metabolism [35].

In many ways, running the *Yale Scientific Magazine* in college was not that different from being Editor-in-Chief of the *Journal of Lipid Research* except in size, scope, and budget. In both cases, articles were both submitted and solicited, there had to be enough articles for a full monthly press run, an attractive cover and theme needed to be designed, and the budget had to be balanced. I was pleased with our success in increasing the scientific impact of the *Journal of Lipid Research* during my tenure and especially that when I stepped down as Editor-in-Chief, the journal had a sizable endowment (USD 2M) to ensure its long term independence. Most importantly, one had to learn to facilitate an interactive and productive editorial team of experts to produce a scientifically interesting and inspiring monthly issue of interest to the readership. One advantage for me was that in the case of the *Yale Scientific Magazine* and in the early days of the *Journal of Lipid Research*, the Editor did not have to worry about “impact factors”. However, over my tenure with ASBMB and its journals, I became intimate with the transformation to online publications with open access and the challenges and negative effects of impact factor ratings on academic publishing.

The final NIGMS progress report for LIPID MAPS reported over 400 publications. It stated that this “demonstrates conclusively that our systems biology approach to defining the many thousands of lipid molecular species in cells and tissues experiencing stress and challenge has already led to many new insights into metabolism, and will continue to lead to breakthroughs in the understanding and treatment of lipid-based diseases”. Additionally, co-investigators of the LIPID MAPS Consortium organized special lipidomics-focused issues for various journals and served as associate editors, editorial board members, reviewers, and authors for the *Journal of Lipid Research.* These experiences enriched all of us intellectually and gave us a broader perspective to the global lipidomics efforts that contributed to the outreach and publications of LIPID MAPS.

### 3.3. Outtake: Scientific Conferences

I first attended a scientific conference when I was in high school and my uncle (Dr. Bernard M. Marks), who was a research scientist at DuPont, visited Chicago to attend the American Chemical Society National Meeting and invited me to accompany him one afternoon. What interested me more than the conference lectures was walking through the exhibition hall, seeing all the “chemical” instruments, and “playing” with the gadgets. The first significant conference I attended was the Lipids Gordon Research Conference (GRS) at Kimball Union Academy in Meriden, New Hampshire, in 1969 as a postdoc. It was an opportunity to interact one on one with the leaders in the field. I particularly enjoyed discussions over drinks at the bar with Nobel Laureate Konrad Bloch (who determined how cholesterol is biosynthesized) and Roy Vagelos, who was the Chair of the Conference and then Chair of Biochemistry at Washington University. Vagelos later became CEO of Merck Pharmaceuticals after leading the development of statin drugs for cardiovascular disease. I subsequently attended numerous other GRCs, was organizer and Chair of the 1989 Lipid Metabolism GRC and then served on the GRC Site and Selection Committee. Later, I served as Chair of the Board of Directors of the Gordon Research Conferences when it was holding over 300 conferences per year. During that time, we established with the Executive Director Dr. Nancy Gray a long-term plan that would ensure the sustainability of the GRC with a formidable endowment by its 100th anniversary.

At the 1969 GRC, I also met Professor C. Fred Fox, who later invited me to participate in the first California Membrane Conference, which he organized at Squaw Valley, CA, USA, in February 1972. There, I enjoyed skiing with participants and discussing the latest science on the ski lifts on the way up the mountain. I also recruited one my earliest postdocs during the poster session. This turned out to be the first conference of what is now known as the Keystone Symposia on Cellular and Molecular Biology; it is currently (2022) celebrating its 50th anniversary. Over the years, I attended many Keystone Symposia and I organized and chaired the first one on signal transduction in 1988 for which Michael Berridge, Tony Hunter, and Robin Irvine joined me as co-chairs. This led to my organizing two more Keystone Symposia on this subject (1994 and 1998), which put me in close contact with the leaders in this then emerging field in which lipids play such a key role and motivated me to explore new directions and connections between lipid metabolism and cell signaling.

That first “skiing symposium”, held in 1972, spawned a sizable organization holding interdisciplinary conferences across the fields of cell, molecular and membrane biology, immunology, protein structure/function, and cancer. In 1990, the organization became part of the Keystone Center, a nonprofit headquartered in Keystone, Colorado, focused on environmental issues and science education at all levels. In 1996, the Keystone Symposia became an independent 501(c)_3_ non-profit solely devoted to organizing scientific conferences in the biomedical sciences. I was elected President and Chair of the Board of Directors of the newly independent organization, a position I held for eight years during which we established a sizable endowment to ensure a sustainable future. Subsequently, I was elected an emeritus trustee, which has allowed me to continue actively in the organization to this day. I am proud of the evolution of the Keystone Symposia and its leadership in organizing some 50–60 international conferences each year that cover the landscape of the exciting and new areas in the biomedical sciences, including some of the top lipid metabolism and lipidomics conferences over the years.

It seems that scientific conferencing organizations, rather than being proprietary, similar to commercial enterprises, are more cooperative and fluid. In 1988, Professor Mosely Waite (Bowman Gray Medical School, Wake Forest University, Winston-Salem, NC, USA) and I initiated a series of FASEB conferences on phospholipases. They have continued to this day with the latest (2022) under the title of “The Phospholipids Conference: Dynamic Lipid Signaling in Health and Disease.” These conferences stimulated the development of the phospholipase A_2_ field, and it was through discussions at these conferences that I developed the Group Numbering System for phospholipase A_2_ enzymes.

A required component of the LIPID MAPS Initiative was holding an annual conference open to the lipidomics community. My previous experience with conferences aided me enormously in organizing these annual conferences. They were a wonderful outreach to other scientists working in the field and included lectures by LIPID MAPS co-investigators and lipid scientists from around the world, including active participation by numerous graduate and postdoctoral students. A special feature was that the annual symposia provided an opportunity to educate scientists and students on how to use the LIPID MAPS website, including the LIPID MAPS Structural Database and other tools it developed. This conference was so successful that when the LIPID MAPS initiative completed its eleven-year lifetime, the NIH granted us a special supplementary conferencing grant to continue the series for an additional three years. Similarly, NIH granted the Bioinformatics Core, under the direction of Professor Shankar Subramaniam, a special three-year supplementary grant to continue its operation. These two three-year grants provided the essential transition time needed for the Wellcome Trust in the UK to fund the continuation of the LIPID MAPS website and databases, allowing the initiative to continue (15). Fortunately, both the Gordon Research Conferences and The Keystone Symposia took up the mantle and organized successful lipidomics meetings that will hopefully continue into the future.

## Data Availability

Not applicable.
